# Documenting Mantodea species in South African museum collections and an updated species list

**DOI:** 10.3897/BDJ.11.e102637

**Published:** 2023-12-12

**Authors:** Bianca Greyvenstein, Johnnie van den Berg, Hannalene du Plessis

**Affiliations:** 1 North-West University, Potchefstroom, South Africa North-West University Potchefstroom South Africa

**Keywords:** diversity, mantids, museum, species and South Africa

## Abstract

**Background:**

The previous species list of South African Mantodea, published in 1998, was largely compiled from the literature and did not incorporate data from the many insect museum collections available in the country. It is estimated that approximately 120 species of Mantodea occur in South Africa; however, since no historical museum records were previously incorporated, the current information is considered to be outdated and not a true reflection of the Mantodea fauna within this region. A checklist of species is an important benchmark for any insect group, especially in light of the worldwide declines of insect diversity reported over the last decade. Checklists that provide accurate information on insect diversity, especially for groups, such as the Mantodea which could be under threat and thus could provide important information that can be used in determining the threat status of species, as well as to aid in their conservation in general.

**New information:**

This paper provides an updated checklist of the praying mantids (Insecta, Mantodea) species of South Africa. While 120 species were previously reported to occur in South Africa, this paper reports 157 species in 64 genera that represent eight different superfamilies, 14 families and 22 subfamilies. Additionally, five species are reported for the first time to occur in South Africa. This species list was generated from the approximately 4000 specimen records of which 3558 records reside within South Africa. The remaining 732 records represent 14 other African countries. Occurrence records from two citizen-science platforms (iNaturalist and Gbif.org), were also incorporated in this study, adding 1880 species records in South Africa. The low number of specimens in the national collections indicate that this group of insects is poorly collected and highlights the lack of knowledge about South Africa’s mantid fauna, as well as a lack of taxonomic expertise as 1532 museum specimens remain unidentified to species level.

## Introduction

Until recently, the Mantodea Order consisted of approximately 24 families with 2400 species (Wieland and Schutte 2012, McMonigle 2013, Wieland 2013, Green 2014). The classification system has recently been revised and the Mantode Order now composed of 16 superfamilies, 29 families and 436 different genera (Schwarz and Roy 2019).

Approximately 120 species of Mantodea were reported to occur in South Africa (Schoeman 1985a, Schoeman 1985b), when the previous species list was compiled between 1996 and 1998 (Kaltenbach 1996, Kaltenbach 1998). Kaltenbach (1996) estimated that there were approximately 131 Mantodea species in South Africa and that 19% of these species were endemic. Ehrmann (2002) estimated a total of 125 species within the region. Beyond the abovementioned checklist information, very little is known of South African Mantodea biology and ecology. A 2023 Scopus (www.scopus.com) internet search of published scientific papers indicated that between 1927 and 2023, 792 papers were published on Mantodea worldwide. However, only 15 of these publications were from institutions in South Africa, seven of which belong to the authors of this paper and two papers were the previous checklist from Kaltenbach (1996), Kaltenbach (1998). The remaining papers conducted on Mantodea in South Africa all addressed molecular and genetic aspects and, in some of these cases, it was actually Blattodea that were investigated. Studies on the biology and distribution of mantids throughout the world are limited and, in South Africa, largely absent.

It is possible that many mantid species in the southern African region have not been documented yet. The only surveys of Mantodea in South Africa were done by Kaltenbach (1996) and the Mantodea Project which was done in collaboration with the Cleveland Museum of Natural history in Ohio, USA (Svenson et al. 2012). However, no species list from the latter survey was published. The latter survey was done during 2005 and only included three regions within South Africa (Cape floristic region, Richards Bay in KwaZulu-Natal Province, and the Kruger National Park in Mpumalanga Province).

This paper, compiled from museum records and previous checklists by Kaltenbach (1996), Kaltenbach (1998), contributes to the information on Mantodea in South Africa and identifies the knowledge gaps with regards to mantids in South Africa.

## Materials and methods

**Figure 1.**
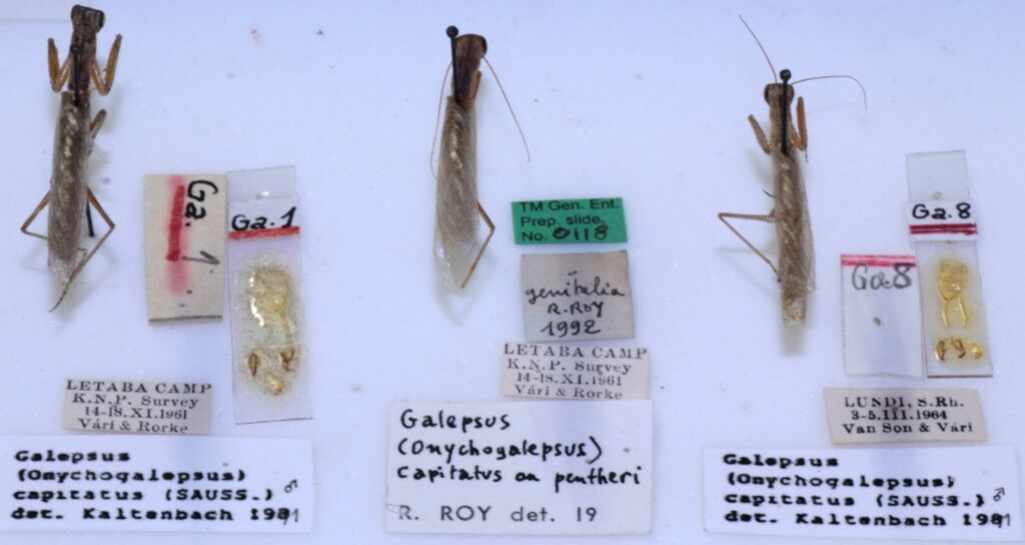
An example of Mantodea museum specimens in a collection which were identified (including genitalia plates if applicable per species) by various taxonomists. These specimens were amongst those used to compile the species list in this paper.

All of the National insect collections and museums throughout South Africa were visited during this study. The following seven institutions constitutes the national insect collections in the country: Ditsong Museum of Natural History (Pretoria) (DNMNH), Agricultural Research Council (Biosystematics Division, Pretoria) (ARC), National Museum (Bloemfontein) (NMB), Albany Museum (Makhanda) (AMG), Rhodes University (Makhanda) (RU), Durban Natural Science Museum (DNSM), Iziko South African Museum (Cape Town) (Iziko) and KwaZulu-Natal Museum (Pietermaritzburg) (NMSA). Specimens in these collections where mostly identified by visiting taxonomists during previous visits to these institutions. Many of the museum specimens were also previously identified by taxonomists at the departments of Dr. Max Beier at the Vienna museum in Germany, Dr. James Rehn at the Academy of Natural Sciences in Philadelphia, USA, Dr. Alfred Peter Kaltenbach at the Natural History Museums in Wien, Austria and Dr. Roger Roy at the Muséum national d’Histoire naturelle (MNHN) in France (Fig. 1).

Furthermore, a small subset of South African Mantodea were identified by Nicolas Moulin, Honorary Associate at Muséum national d'Histoire naturelle in France, during 2019. Unidentified specimens that were encountered in the abovementioned museums were identified by means of the literature and through assistance from a taxonomist who specializes in African Mantodea (Nicolas Moulin). Many Mantodea specimens in South African collections have only been identified to genus level. These "ignota specimens" (approximately 1600) were, therefore, not included in this checklist. However, they are included in the database itself (available in Suppl. material 1).

In order to compile this database, all of the Mantodea specimens and distribution labels were photographed and the label information documented. This database contains the following information for each specimen record: genus and species name, collector’s details, collection date, if available, and locality. The website mantodeaspeciesfile.org (Otte et al. (2022) as well as other literature on specific species, such as those by Ehrmann (2002), Roy (2004), Roy (2006), Roy (2009), Roy (2010), Roy (2013), Roy (2018), Roy (2022) were used to determine the current nomenclature. The reclassification of the Order Mantodea by Schwarz and Roy (2019) was also applied during the updating of this checklist. The updated species list was compared to that provided in publications by Kaltenbach (1996), Kaltenbach (1998) after which similarities and differences were highlighted.

To our knowledge, this paper provides the most comprehensive list of Mantodea in South African collections. Since only a limited number of Mantodea specimens of 14 other African countries were present in South African museum collections, these records were not included in this paper. The scope of this study, did however not allow for vistits to museums residing outside of South Africa, which is required when the latter information is used to compile comprehensive Mantodea species lists for these African countries. However, to increase the comprehensiveness of this checklist, various European and American museum collections were contacted and provided information on South African Mantodea in their collections. These museums were: The Natural History Museum, (Italy); Smithsonian National Museum of Natural History (USA), Museum für Naturkunde (Germany), Natural History Museum (UK), Staatliches Museum für Naturkunde (Germany), Royal Belgian Institute of Natural Sciences (Belguim) and the Muséum national d’Histoire naturelle (France). Data from Mantodea specimens in a private collection in Germany (Christian Schwarz), as well as records relevant Gbif.org records within South Africa were also included in this study.

This species list includes information on the taxonomists who identified the species in the South African museum collections and is indicated for each species by the “ID” tag, as well as the year in which the specimen was identified (if available). Furthermore, the hosting museum collection of each specimen is also included in brackets (). A list of abbreviations for the various institutions and collections are provided in Table 1. Specimens that are not held locally, or for which only literature records exist, are indicated under the ID tag column in the checklist as with the abbreviation "Lit" with the reference to the relevant publications. The number of records in the various collections in South Africa, as well as the number of Research Grade observation records of the species listed on the two citizen-science platforms, are provided in Suppl. material 1. It should be noted that no details of the persons who provided identifications of species listed on the citizen-science platforms are listed in the checklist. This will be addressed in the Discussion section of the paper.

The geographical distribution of species beyond South Africa, is based on information provided by Roy (1967), Kaltenbach (1996), Kaltenbach (1998), Ehrmann (2002), Roy (2004), Roy (2006), Roy (2009), Roy (2010), Roy (2013), Roy (2018), Roy (2022) as well as museum records indicated below, after the tag "Distribution". The abbreviations used for the different countries are listed in Table 2. It should be noted that all the species listed below are present in South Africa and, thus, this country is not listed under the distribution. Where there is no “Distribution” tag associated with a species, the species has only been reported from South Africa. The specimen type, i.e., Holotype, Paratype or DNA barcode, sex, and museums in which they are kept, is provided in the Suppl. material 1.

### Results

**Figure 2.**
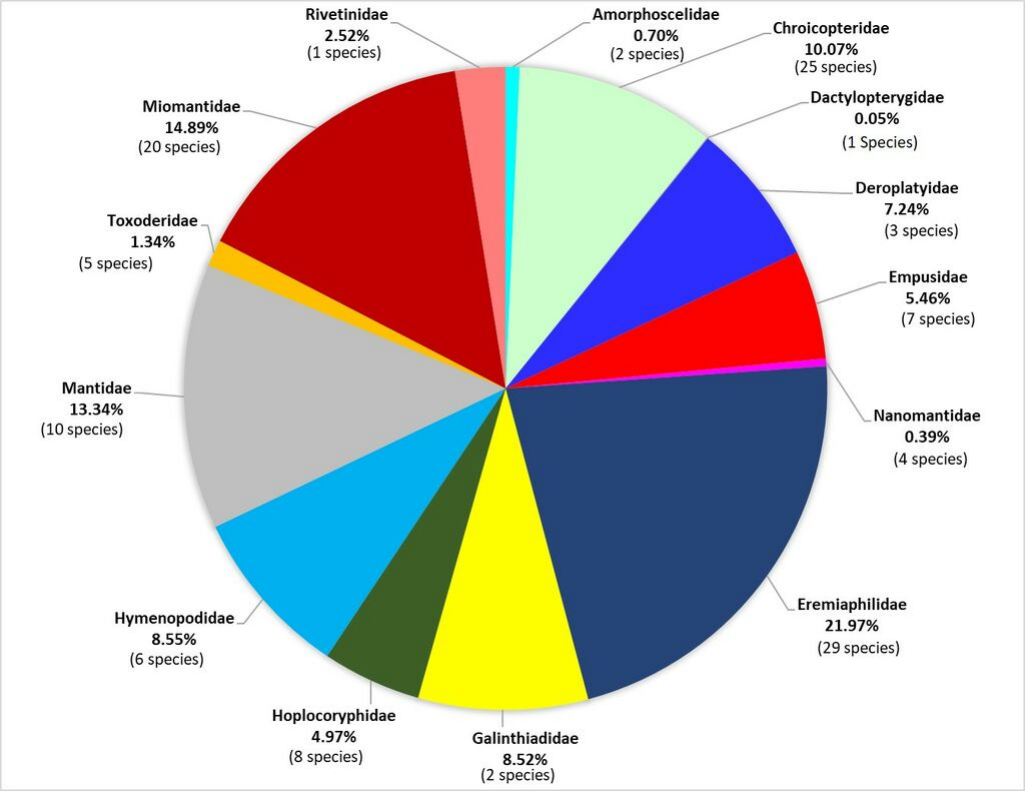
Summary of the number of species and number of records per Mantodea family recorded in South Africa.

This updated checklist includes information on species of the Mantodea that were not previously listed in South African checklists. The known species richness has increased from approximately 120 species in 1998 to 157 species (this report). The South African Mantodean fauna have eight superfamilies, 14 families, 22 subfamilies, 19 tribes, 14 subtribes and 15 genera (Suppl. material 2). A summation of the number of records and species within these 14 families is presented in Fig. 2.

This checklist encompasses 157 Mantodea species that occur, or are reported to occur in South Africa, including the first report of five species within the region (indicated with two asterisks ** in the notes section of each species). However, some anomalies were recorded (indicated by # in the notes section of each species). These anomalies are addressed in the Discussion section of this paper.

## Checklists

### Updated checklist of Mantodea of South Africa

#### Chroicopteroidea

Giglio-Tos, 1915

B5005E4B-D32A-52D1-9DA4-73A74F268369

#### Chroicopteridae

Giglio-Tos, 1915

6F5CF9BB-B5D3-55F7-9DC6-6C78E2AA436C

#### Chroicopterinae

Giglio-Tos, 1915

BD72CE91-AA1C-5161-8E4F-3BBAFB831D0F

#### Bolbellini

Schwarz & Roy 2019

4C782927-0D76-55A6-BC84-9BE474730DFD

#### Dystactula grisea

Giglio-Tos, 1915

9C65F3B0-2C40-5B33-98FF-E89A3608A908

##### Native status

Suspected to be endemic to southern Africa (Kaltenbach 1996)

##### Distribution

MOZ

##### Notes

ID: N. Moulin 2018. (DNMNH)

#### Dystactula natalensis

Kaltenbach, 1996

515B8FBB-9CA5-5201-957A-B93EAC41F6B8

##### Native status

Suspected to be endemic to southern Africa (Kaltenbach 1996)

##### Notes

ID: Lit (Kaltenbach 1996, Ehrmann 2002)

#### Chroicopterini

Giglio-Tos, 1915

2E12AA7B-F6FE-5662-ABE1-E2E6DDF5DCC1

#### Bisanthina

Giglio-Tos, 1917

45E41E94-DCC4-5783-A44E-A6403CDE1E89

#### Bisanthe lagrecai

Kaltenbach, 1996

0D7A9426-5F3C-59A3-A346-F882A4E8A633

##### Native status

Suspected to be endemic to southern Africa (Kaltenbach 1996)

##### Distribution

ZIM

##### Notes

ID: Dep. A. Kaltenbach 1989. (DNMNH)

#### Bisanthe pulchripennis

(Stål ,1876)

A8BE1481-F2DD-5C61-A0F0-CC6CDF2CFA5E

##### Native status

Suspected to be endemic to southern Africa (Kaltenbach 1996)

##### Distribution

NAM, BOT

##### Notes

ID: Dep. J.A.G. Rehn 1925, A. Kaltenbach 1992;1989. (DNMNH, IZIKO)

#### Bolbellini

Schwarz & Roy 2019

B27D6C85-ACFC-5FCA-B67B-ED916E5912A7

#### Bolbella affinis

Kaltenbach, 1996

5F933481-E7D4-50BA-AD2D-2E4F33AF7896

##### Notes

ID: Lit (Kaltenbach 1996, Ehrmann 2002

#### Bolbella brevis

Beier, 1953

698C40DE-D2E9-5649-B256-7BD85D923182

##### Native status

Suspected to be endemic to southern Africa (Kaltenbach 1996)

##### Notes

ID: Unspesified. (DNMNH)

#### Bolbella punctigera

(Stl, 1871)

5B11377F-2EFF-5CB3-AC9D-EF9C151DC5F9

##### Native status

Suspected to be endemic to southern Africa (Kaltenbach 1996)

##### Distribution

CA, LS

##### Notes

ID: Dep. J.A.G. Rehn 1925 & A. Kaltenbach 1982. (BOLD, DNMNH, IZIKO)

#### Bolbella rhodesiaca

Beier, 1930

C2EF4EFC-A0A9-5B7C-92FD-505ED2F04F70

##### Native status

Suspected to be endemic to southern Africa (Kaltenbach 1996)

##### Distribution

ZIM

##### Notes

ID: Dep. H.D. Brown 1963, M. Beier 1963. (ARC, DNMNH)

#### Chroicopterina

Giglio-Tos, 1915

6CE97810-BB8F-5A8F-BD14-5304F6791492

#### Carvilia gracilis

Kaltenbach, 1996

97D5F927-6000-5502-93AB-5213D223250E

##### Native status

Suspected to be endemic to southern Africa (Kaltenbach 1996)

##### Notes

ID: Lit (Kaltenbach 1996, Ehrmann 2002)

#### Chroicoptera saussurei

(Giglio-Tos, 1915)

127F4F21-C037-5514-B70C-46C939B62569

##### Distribution

LS

##### Notes

ID: Dep. A. Kaltenbach 1989 & A.J. Hesse. (DNMNH, IZIKO)

#### Chroicoptera vidua

(Stål, 1856)

17E59EA3-DD35-58D8-8B58-26050DFAC3FF

##### Distribution

NAM

##### Notes

ID: Dep. J.A.G. Rehn 1925. (DNMNH)

#### Chroicoptera longa

Giglio-Tos, 1915

73500BB0-D49F-5381-88CF-DCB053AA9C3A

##### Notes

ID: Lit (Kaltenbach 1996)

#### Entella (Entella) delalandi

(Saussure, 1870)

49EBF620-8DC8-52D5-9366-819494A084C9

##### Native status

Suspected to be endemic to southern Africa (Kaltenbach 1996)

##### Distribution

NAM, TZ, ZIM

##### Notes

ID: Dept. A. Kaltenbach 1989, M. Beier 1925, R. Erhmann & F. Werner. (DNMNH, NRM, SMNK)

#### Entella (Entella) exilis

Giglio-Tos, 1915

495CB620-1315-56C9-BEAC-0E39F6D511CF

##### Native status

Suspected to be endemic to southern Africa (Kaltenbach 1996)

##### Notes

ID: Lit (Ehrmann 2002)

#### Entella (Entella) natalica

Beier, 1955

39FC13AE-3CC3-5AAF-9036-31C432C7CEDA

##### Native status

Suspected to be endemic to southern Africa (Kaltenbach 1996)

##### Notes

ID: Dep. M. Beier 1953. (MZLU)

#### Entella (Entella) nebulosa

(Audinet-Serville, 1839)

AC2146D1-62B0-5AD9-B421-B9006A1058A6

##### Native status

Suspected to be endemic to southern Africa (Kaltenbach 1996)

##### Notes

ID: Dep. A.J. Hesse. (IZIKO, NRM).

#### Entella (Entella) orientalis

Giglio-Tos, 1915

E72D47B8-167A-59CC-B8EB-46B56D90C45C

##### Distribution

MOZ, TZ

##### Notes

ID: Lit (Kaltenbach 1996, Ehrmann 2002)

#### Entella (Entella) pusilla cruciata

Beier, 1953

1F549696-132B-5161-85A7-29372F0CF795

##### Notes

ID: Dep. M. Beier 1952. (DNMNH)

#### Entella (Entella) pusilla pusilla

Beier, 1953

695B20E5-AA22-5A25-9637-3CAEC945AB46

##### Notes

ID: Dep. M. Beier 1952. (DNMNH).

#### Entella (Entella) rudebecki

Beier, 1955

5356E002-0E1C-501F-808B-6CCA09F9FB02

##### Native status

Suspected to be endemic to southern Africa (Kaltenbach 1996)

##### Distribution

LS

##### Notes

ID: Lit (Ehrmann 2002)

#### Entella (Entella) taborana

(Giglio-Tos, 1915)

4D3948A8-F4F3-590C-B589-B7A05FFBAC73

##### Distribution

TZ

##### Notes

ID: Dep. M. Beier 1952. (DNMNH) **

#### Entella (Entella) transvaalica

Beier, 1955

4C15B08F-032E-5B5B-BCC9-D4E44A38BFA2

##### Native status

Suspected to be endemic to southern Africa (Kaltenbach 1996)

##### Distribution

ZIM

##### Notes

ID: Dep. A. Kaltenbach 1985 & R. Ehrmann. (DNMNH, SMNK)

#### Entelloptera rogenhoferi rogenhoferi

Saussure, 1872

88ACD66B-775B-5BFE-AD21-1C5C85F6A647

##### Distribution

NAM, TZ, ZIM

##### Notes

ID: Dep. A. Kaltenbach 1984;1991 & M. Beier 1952. (ARC, DNMNH, IZIKO)

#### Entelloptera rogenhoferi maesta

Rehn, 1927

A79FE59C-C0C2-5CD7-8F90-5FFB328C90D8

##### Native status

Suspected to be endemic to southern Africa (Kaltenbach 1996)

##### Notes

ID: Dep. J.A.G. Rehn 1925. (DNMNH)

#### Geothespis australis

Giglio-Tos, 1916

7C00528B-56CF-5327-9C08-BFA4FFF28738

##### Native status

Suspected to be endemic to southern Africa (Kaltenbach 1996)

##### Distribution

AZ, NAM

##### Notes

ID: Lit (Kaltenbach 1996)

#### Ligaria affinis

Kaltenbach, 1996

A2E9003C-EDAF-57CD-BF28-51C7DC6FC5D5

##### Distribution

BOT, MOZ, ZAM, ZIM

##### Notes

ID: Lit (Ehrmann 2002)

#### Ligaria brevicollis

Stål, 1877

9855CE40-4C40-5C4D-AFA5-E288252A5506

##### Native status

Suspected to be endemic to southern Africa (Kaltenbach 1996)

##### Distribution

MOZ, ZIM

##### Notes

ID: Dep. M. Beier 1952, A. Kaltenbach 1985 & N. Moulin 2018. (DNMNH, NRM)

#### Ligaria quadrinotata

Chopard, 1914

98CB5993-9636-5231-A5E9-7EF0B76A8C26

##### Distribution

NAM

##### Notes

ID: Dep. A. Kaltenbach. (NRM)

#### Ligariella gracilis

Karny, 1908

80EC1724-5515-5080-BBB6-6FAC93EF1619

##### Native status

Suspected to be endemic to southern Africa (Kaltenbach 1996)

##### Notes

ID: Lit (Ehrmann 2002)

#### Ligariella trigonalis

Saussure, 1899

5965E004-7463-5F2F-A472-FB9325E3EAFC

##### Native status

Suspected to be endemic to southern Africa (Kaltenbach 1996)

##### Distribution

BOT, NAM

##### Notes

ID: Dep. M. Beier 1952 & A. Kaltenbach 1985. (DNMNH, IZIKO)

#### Namamantis cruciata

Beier, 1953

F71E0F55-7009-545F-8709-74B941570613

##### Native status

Suspected to be endemic to southern Africa (Kaltenbach 1996)

##### Notes

ID: Lit (Kaltenbach 1996, Ehrmann 2002) & (PC_CS)

#### Namamantis nigropunctata

Kaltenbach, 1996

169DAD88-8CD2-5659-A86D-D3908F61ACF8

##### Native status

Suspected to be endemic to southern Africa (Kaltenbach 1996)

##### Notes

ID: Lit (Kaltenbach 1996, Ehrmann 2002) & (PC_CS)

#### Dystactina

Giglio-Tos, 1915

5693B03E-586F-529F-9CCA-DA3138FFC6A9

#### Dystacta alticeps

(Schaum, 1852)

D049B328-8FD2-52F5-A652-343BD9F1451A

##### Distribution

AG, BOT, DRC, MAL, MOZ, NAM, TZ, ZIM

##### Notes

ID: Dep. J.A.G. Rehn 1925, M. Beier 1952, R. Roy 1977, A. Kaltenbach 1984, R. Erhmann & N. Moulin 2018. (ARC, DNMNH, IZIKO, NMSA, SMNK, PC_CS)

#### Tarachininae

Giglio-Tos, 1915

B486A2D3-B4BE-5B3D-BB67-62929B6BE4B3

#### Gonypetella atrocephala

Beier, 1930

7EC03200-F977-512B-BF8A-0E434932CA68

##### Native status

Suspected to be endemic to southern Africa (Kaltenbach 1996)

##### Notes

ID: Lit (Ehrmann 2002)

#### Gonypetellini

Schwarz & Roy, 2019

8B049979-8F14-5DF6-B2A1-5DE650662CFF

#### Gonypetella deletrix

Rehn, 1927

0B3103F8-6FA1-5855-824E-1DEA44C28E21

##### Native status

Suspected to be endemic to southern Africa (Kaltenbach 1996)

##### Distribution

AG, NAM, ZIM

##### Notes

ID: Dep. J.A.G. Rehn 1925, A. Kaltenbach 1989, A.J. Hesse, C. Schwarz & R. Erhmann. (DNMNH, IZIKO, SMNK, PC_CS)

#### Gonypetella punctata

Giglio-Tos, 1915

55B0AFDB-EB7F-51A9-8903-CC08E2665B7D

##### Distribution

CAM, KN, DRC, UG

##### Notes

ID: Undefined. (NRM)

#### Tarachinini

Giglio-Tos, 1915

64940382-5116-5574-803B-E4E31058633C

#### Tarachina schultzei

Karny, 1908

B16B745F-757C-5A3E-A427-681ED54CA67E

##### Native status

Suspected to be endemic to southern Africa (Kaltenbach 1996)

##### Distribution

NAM, ZIM

##### Notes

ID: Dep. J.A.G. Rehn 1925. (DNMNH, IZIKO)

#### Tarachina transvaalensis

Beier, 1953

5DE57ADA-9D1A-5A7B-9259-B262747CDB7C

##### Native status

Suspected to be endemic to southern Africa (Kaltenbach 1996)

##### Distribution

ZIM

##### Notes

ID: Dep. M. Beier 1952, A. Kaltenbach 1989, R. Ehrmann 1991 & A. Kaltenbach 1992 (ARC, DNMNH)

#### Eremiaphiloidea

Saussure, 1869

F5F4D540-8BB6-5F93-9754-0D3D13C58412

#### Eremiaphilidae

Saussure, 1869

9AEB53CC-9EBF-5322-B7ED-4426CD339442

#### Iridinae

Westwood, 1889

C0EF841D-3608-540C-849E-9CCF21F1E251

#### Iridini

Westwood, 1889

B9AC65DD-986F-5A53-A936-80FCFBDF6394

#### Episcopomantis chalybea

Burmeister, 1838

DF8561AD-3742-59B9-9A26-27C51C519F04

##### Distribution

AG, BOT, NAM

##### Notes

ID: Dep. R. Roy 1977, A. Kaltenbach 1984, A.J. Hesse, M.B.D. Stiewe 2003 & R. Ehrmann. (ARC, DNMNH, IZIKO, SMNK)

#### Tarachodinae

Giglio-Tos, 1915

4F6700C6-4E22-55A2-A38F-B0914B57D3D7

#### Oxyelaeini

Schwarz & Roy 2019

44676B51-D990-5935-8A63-3F3B394F3B97

#### Oxyelaea elegans

Giglio-Tos, 1917

E8E63A0F-1F87-5FEB-BE79-7866CA3F204E

##### Distribution

DRC, TZ

##### Notes

ID: Dep. M.B.D. Stiewe & N. Moulin 2018. (ARC, NMSA, NMB) **

#### Tarachodini

Giglio-Tos, 1915

242C4F5D-9AA3-532A-A534-CE6E2978542E

#### Antistiina

Schwarz & Roy 2019

49256DD1-840C-5836-B9DB-4E051FDAF268

#### Antistia maculipennis

Stål, 1876

7BCDA125-9B1E-5F89-B13F-2C826EDF44F2

##### Distribution

BOT, NAM, TZ

##### Notes

ID: Dep. M. Beier 1952, R. Roy 1962, A. Kaltenbach 1984 & F. Werner. (ARC, DNMNH, IZIKO, NRM)

#### Antistia parva

Beier, 1953

16D6A131-B366-507A-8D64-7617FF251663

##### Distribution

NAM

##### Notes

ID: Dep. M. Beier 1952 & A. Kaltenbach 1989. (DNMNH)

#### Antistia robusta

Kaltenbach, 1996

0FF1BEC1-EA89-5C54-8FC9-57270470A106

##### Distribution

TZ

##### Notes

ID: Dep. A. Kaltenbach 1989. (DNMNH)

#### Antistia vicina

Kaltenbach, 1996

768E2FB5-7858-5CCC-8861-07296F70B752

##### Distribution

NAM

##### Notes

ID: Dep. A. Kaltenbach 1989 (DNMNH, PC_CS)

#### Ariusia conspersa

Stål, 1877

F2CEE60C-3A06-5414-9630-0B37E5EF091B

##### Distribution

NAM, ZIM

##### Notes

ID: N. Moulin 2018. (NMB)

#### Tarachodina

Giglio-Tos, 1915

01883466-0813-526C-88B4-3F413FEF8196

#### Pyrgomantis fasciata

Giglio-Tos, 1917

D6B61E60-1734-556D-99B3-B5E212E697BB

##### Notes

ID: Undefined. (DNMNH).

#### Pyrgomantis nasuta

Thunberg, 1784

C48EB4CC-D8D9-591A-B5C6-2A459FA7BCBE

##### Distribution

AG, CAM, KN, NAM, SM, TZ

##### Notes

ID: Dep. J.A.G. Rehn 1925, A. Kaltenbach 1984, R. Ehrmann 1991 & A. Kaltenbach 1992. (ARC, DNMNH, IZIKO, NRM).

#### Pyrgomantis rhodesica

Giglio-Tos, 1917

A0F340F3-CE5F-5630-BB14-3299451BB73C

##### Native status

Suspected to be endemic to southern Africa (Kaltenbach 1996)

##### Distribution

BOT, NAM, ZAM, ZIM

##### Notes

ID: Dep. M. Beier 1952 & A. Kaltenbach 1984; 1992. (ARC, DNMNH)

#### Pyrgomantis simillima simillima

Beier, 1954

FB3B87B8-4553-5205-9A22-799FBDA691A5

##### Native status

Suspected to be endemic to southern Africa (Kaltenbach 1996)

##### Distribution

ZIM

##### Notes

ID: Dep. A. Kaltenbach 1992. (DNMNH)

#### Galepsus (Lygdamia) lenticularis

(Saussure, 1872)

439A5EA0-327D-53AE-95B4-628BC2BD5574

##### Native status

Suspected to be endemic to southern Africa (Kaltenbach 1996)

##### Distribution

ET, BOT

##### Notes

ID: Dep. M. Beier 1952, A. Kaltenbach 1989, A.J. Hesse, N. Moulin 2018 & R. Ehrmann. (DNMNH, IZIKO, NRM, SMNK)

#### Galepsus (Onychogalepsus) angolensis

Werner, 1907

C575898A-2314-5FAA-ADDA-29E184ACB313

##### Distribution

AG, MOZ

##### Notes

ID: Lit (Ehrmann 2002)

#### Galepsus (Onychogalepsus) capensis

Beier, 1930

16DCD69E-C8E9-5805-AEF2-EBCF2D3CDB4D

##### Native status

Suspected to be endemic to southern Africa (Kaltenbach 1996)

##### Notes

ID: Lit (Ehrmann 2002)

#### Galepsus (Onychogalepsus) capitatus

(Saussure, 1869)

DC7A8CD4-C705-5E8F-8B5B-14D9522BB7AA

##### Native status

Suspected to be endemic to southern Africa (Kaltenbach 1996)

##### Distribution

ET, KN, TZ, ZIM

##### Notes

ID: Dep. A. Kaltenbach 1984;1991. (ARC, DNMNH)

#### Galepsus (Onychogalepsus) centralis

Beier, 1957

D50D6F81-E32F-5064-A937-91D9F922524E

##### Distribution

DRC, TZ

##### Notes

ID: N. Moulin 2018. ^##^

#### Galepsus (Onychogalepsus) damaranus damaranus

Giglio-Tos, 1911

A8372F51-1A2C-5FDE-BE5F-21B2EE3D0F23

##### Distribution

AG, DRC, NAM

##### Notes

ID: Lit (Kaltenbach 1996, Ehrmann 2002)

#### Galepsus (Onychogalepsus) damaranus orientalis

Kaltenbach, 1996

F371E194-73F4-5347-9285-2E7317A191C1

##### Distribution

NAM, ZIM

##### Notes

ID: Lit (Kaltenbach 1996, Ehrmann 2002)

#### Galepsus (Onychogalepsus) femoratus

Giglio-Tos, 1911

175E6620-5CD1-5263-A43E-51F9F4C91B06

##### Native status

Suspected to be endemic to southern Africa (Kaltenbach, 1996)

##### Distribution

NAM, ZIM

##### Notes

ID: Dep. A.J. Hesse. (IZIKO)

#### Galepsus (Onychogalepsus) intermedius

Werner, 1907

69BD947F-AD6F-5C55-9DD0-DB3885817A5B

##### Distribution

KN, MOZ, ZIM

##### Notes

ID: Dep. J.A.G. Rehn 1925, A. Kaltenbach 1991, R. Erhmann 1991, H.D. Brown. (ARC, DNMNH, SMNK)

#### Galepsus (Onychogalepsus) letabaensis

Kaltenbach, 1996

F975D7AE-DADE-5916-A36B-CF89D5780AA4

##### Native status

Suspected to be endemic to southern Africa (Kaltenbach 1996)

##### Notes

ID: Dep. A. Kaltenbach 1992. (DNMNH)

#### Galepsus (Onychogalepsus) meridionalis

(Saussure, 1872)

E73D18BF-D011-50C2-A01B-26E7EE232933

##### Native status

Suspected to be endemic to southern Africa (Kaltenbach 1996)

##### Distribution

DRC, TZ, ZIM

##### Notes

ID: Dep. A. Kaltenbach 1991, A.J. Hesse, B.P. Uvarov, R. Ehrmann & F. Werner. (DNMNH, IZIKO, NMSA, SMNK, NRM)

#### Galepsus (Onychogalepsus) pentheri

Giglio-Tos, 1911

612F1C88-FD1D-51C8-A89F-29ECFE66BD16

##### Native status

Suspected to be endemic to southern Africa (Kaltenbach 1996)

##### Distribution

CAM, DRC, NAM (possibly)

##### Notes

ID: Dep. A. Kaltenbach 1984; 1991 & C. Schwarz. (ARC, DNMNH, PC_CS)

#### Galepsus (Onychogalepsus) transvaalensis

Beier, 1954

93AC1827-6B81-5247-80FB-5D4A428CE909

##### Native status

Suspected to be endemic to southern Africa (Kaltenbach 1996)

##### Distribution

DRC

##### Notes

ID: Dep. A. Kaltenbach 1984; 1989; 1992. (DNMNH)

#### Galepsus (Syngalepsus) beieri

Kaltenbach, 1996

A05CFC53-D220-586B-A854-33260B72E5EB

##### Native status

Suspected to be endemic to southern Africa (Kaltenbach 1996)

##### Notes

ID: Lit (Kaltenbach 1996, Ehrmann 2002)

#### Galepsus (Syngalepsus) bipunctatus

Beier, 1931

1E7AB4CC-A8C4-5ECC-A54B-1B949746E260

##### Distribution

MOZ

##### Notes

ID: Dep. A. Kaltenbach 1991 & R. Erhmann. (DNMNH, SMNK)

#### Nothogalepsus planivertex

Beier, 1969

AEB793A3-B9EC-58D3-96A6-EA3700FC0A69

##### Distribution

MAL, MOZ, NAM, ZIM

##### Notes

ID: Dep. A. Kaltenbach 1992. (DNMNH)

#### Tarachodes (Chiropus) dives

Saussure, 1869

8655F2EE-3F51-5771-90CD-9ADC78CEFB72

##### Native status

Suspected to be endemic to southern Africa (Kaltenbach 1996)

##### Distribution

AG, NAM, SD

##### Notes

ID: Dep. A. Kaltenbach 1992. (DNMNH) **

#### Tarachodes (Tarachodes) beieri

Kaltenbach, 1996

64E61715-E2AB-5682-8035-EA4ABED0A74F

##### Native status

Suspected to be endemic to southern Africa (Kaltenbach 1996)

##### Distribution

ZIM

##### Notes

ID: Dep. A. Kaltenbach 1992. (DNMNH)

#### Tarachodes (Tarachodes) insidiator

Wood-Mason, 1882

34CE5F4C-9C32-50E0-9A3A-193FBF28AFEB

##### Distribution

AG, DRC, KN, MAL, TZ, ZIM

##### Notes

ID: Dep. J.A.G. Rehn 1925, A. Kaltenbach 1989 & B.P. Uvarov. (DNMNH, IZIKO, NMSA)

#### Tarachodes (Tarachodes) lucubrans

Burchell, 1822

400F8A4B-C746-56EA-ADBB-D655F7859B72

##### Notes

ID: Dep. M. Beier 1952, R. Roy 1977 & A. Kaltenbach 1989. (ARC, DNMNH, IZIKO, NMSA)

#### Tarachodes (Tarachodes) maurus

Stål, 1856

4247D51E-A666-5246-811E-AEB22DA75B8C

##### Distribution

CAM, MAL, MOZ, NAM, TZ

##### Notes

ID: Dep. M. Beier 1952 & F. Werner. (DNMNH, IZIKO, NRM)

#### Tarachodes (Tarachodes) sanctus sanctus

Saussure 1871

864F4628-74E2-52BC-ABDA-C14E192BD055

##### Distribution

DRC, MOZ, SM, TZ, ZIM

##### Notes

ID: Dep. M. Beier 1952 & A. Kaltenbach 1992. (DNMNH, IZIKO, NRM)

#### Tarachodes (Tarachodina) natalensis

Kaltenbach, 1996

B3F217F3-2107-5AE8-9406-28C8049B7513

##### Native status

Suspected to be endemic to southern Africa (Kaltenbach 1996)

##### Notes

ID: Lit (Ehrmann 2002)

#### Rivetinidae

Ehrmann & Roy, 2002

3503CFFA-B26C-5379-80F7-46F1023F21D3

#### Rivetininae

Ehrmann & Roy, 2002

4171D8EF-6D94-5B37-8625-B5239302D387

#### Ischnomantini

Giglio-Tos, 1916

F69FD9AC-519E-5AD5-829D-23482F119D38

#### Ischnomantis fatiloqua

Stål, 1856

70DC6991-FF00-5CCB-85C3-D3E110491CAB

##### Distribution

AG, DRC, NAM, ZIM

##### Notes

ID: Dep. J.A.G. Rehn 1925, R. Roy 1976, R. Ehrmann 1991 & N. Moulin 2018. (ARC, DNMNH, IZIKO, NRM)

#### Toxoderidae

Saussure, 1869

82FB03D8-064E-5602-83B7-483CE1EF0F6A

#### Compsothespinae

Giglio-Tos, 1913

65F32F0F-04A2-59CD-9A56-500D97D66748

#### Compsothespis anomala

Saussure, 1872

7C233FEA-F257-5121-9BB0-755018A7BC02

##### Notes

ID: Dep. M. Beier 1952, H.D. Brown 1963, A.J Hesse & R. Erhmann. (ARC, DNMNH, IZIKO, SMNK)

#### Compsothespis cinnabarina

Beier, 1955

BBCC685E-6A99-5BE3-975B-41738D7461F4

##### Native status

Suspected to be endemic to southern Africa (Kaltenbach 1996)

##### Notes

ID: Dep. M. Beier 1953. (MZLU)

#### Compsothespis kilwana

Giglio-Tos, 1913

007201B4-C1B7-54E5-9FB1-9822B52F1960

##### Notes

ID: Lit (Ehrmann 2002)

#### Compsothespis natalica

Westwood, 1889

1D3F4E90-41B2-5892-9C58-C989984BC005

##### Native status

Suspected to be endemic to southern Africa (Kaltenbach 1996)

##### Notes

ID: Dep. J.A.G. Rehn 1925 & H.D. Brown 1953. (ARC, DNMNH)

#### Heterochaetinae

Brunner de Wattenwyl, 1893

8C424E81-429D-58F1-AEF8-3E3345AAF7D3

#### Heterochaeta occidentalis

Beier, 1963

78E5C516-A8F4-5397-A9CB-3E3403EAD39C

##### Native status

Suspected to be endemic to southern Africa (Kaltenbach 1996)

##### Distribution

BOT, KN, NAM

##### Notes

ID: Dept. R. Roy 1976 & A.J.Hesse. (DNMNH, IZIKO, NMB)

#### Toxoderinae

Saussure, 1869

55BF943B-50A9-593E-941F-DD1E7D2F409A

#### Calamothespini

Giglio-Tos, 1914

A18FBCF2-EA09-58A1-9003-EFBDBA42076E

#### Calamothespina

Giglio-Tos, 1914

A3D14234-C235-5F27-8206-5FFB5B6C4AD6

#### Calamothespis oxyops

Rehn, 1927

29358070-9FD5-5266-BD3C-1E124882EE7B

##### Native status

Suspected to be endemic to southern Africa (Kaltenbach 1996)

##### Notes

ID: Dep. J.A.G. Rehn 1925. (DNMNH)

#### Galinthiadoidea

Giglio-Tos, 1915

134957A2-CFC1-52FB-8256-A1A22D23C71A

#### Galinthiadidae

Giglio-Tos 1919

17815A8B-EA40-53D4-9271-56FE6504D173

#### Galinthias amoena

(Saussure, 1871)

5246AB40-5B49-5C7A-B2DF-267EE3C9A437

##### Distribution

AG, DRC, MAL, TZ, ZAM, ZIM

##### Notes

ID: Dep. R. Roy 1976, A.J.Hesse, C. Schwarz & R. Erhmann. (DNMNH, IZIKO, SMNK, PC_CS)

#### Harpagomantis tricolor

(Linné, 1758)

213E47E4-E353-550B-8FC0-729D796E97DF

##### Distribution

BOT, LS, NAM, ZAM, ZIM

##### Notes

ID: Dep. J.A.G. Rehn 1925, R. Roy 1977, A. Kaltenbach 1984, R. Erhmann 1991, C. Schwarz & B.P. Uvarov. (AMG, ARC, CMNH, DNMNH, IZIKO, PC_CS)

#### Hoplocoryphoidea

Giglio-Tos, 1916

CC79FE87-3EF5-56FC-83C7-4BC11992905D

#### Hoplocoryphidae

Giglio-Tos, 1916

A3FA84DF-1F58-54E8-BBEA-7455DCC71F54

#### Hoplocorypha bicornis

Deelemann-Reinhold, 1957

F1035B4F-523B-5CC5-96B9-70710BA89379

##### Notes

ID: Dep. J.A.G. Gain. (NMB)

#### Hoplocorypha brevicollis

Beier, 1931

7FBEC7E4-322D-50FF-89AB-5A6A5C4B7ABD

##### Native status

Suspected to be endemic to southern Africa (Kaltenbach 1996)

##### Notes

ID: Lit (Ehrmann 2002)

#### Hoplocorypha fumosa

Giglio-Tos, 1916

97665E51-6014-51EA-8937-E4AABF0CD173

##### Distribution

MAL, MOZ, ZIM

##### Notes

ID: Dep. A.J. Hesse. (IZIKO)

#### Hoplocorypha galeata

Saussure, 1870

57C63F8F-BB48-5B53-AF74-E960FCAA3510

##### Distribution

ET, KN, TZ, ZB

##### Notes

ID: Undefined. (NRM)

#### Hoplocorypha macra

Stål, 1856

2C295722-3530-587C-8828-1FFACB1E1A84

##### Native status

Suspected to be endemic to southern Africa (Kaltenbach 1996)

##### Distribution

AG, KN, NAM, TZ, UG, ZAM

##### Notes

ID: Dep. J.A.G. Rehn 1925, M. Beier 1952, A. Kaltenbach 1985 & A.J. Hesse. (NRM, IZIKO, DNMNH, ARC)

#### Hoplocorypha nana

Sjostedt, 1909

334101C9-D0A4-50C4-875D-C2D6C57685BF

##### Distribution

KN, NAM, NG, TZ, UG

##### Notes

ID: Dept. M. Beier 1952 & A Kaltenbach 1985; 1991. (ARC, DNMNH)

#### Hoplocorypha saussurii

Giglio-Tos, 1916

BDE0932F-B5C0-567D-B572-B880B9B6A4DD

##### Distribution

KN, NAM, TZ, ZAM

##### Notes

ID: Dept. M. Beier 1952 & R. Roy 1977. (DNMNH)

#### Hoplocorypha striata

Beier, 1930

D2AFAF0D-BEF3-5D88-90BB-60BA840405EF

##### Native status

Suspected to be endemic to southern Africa (Kaltenbach 1996)

##### Distribution

NAM

##### Notes

ID: Dept. A Kaltenbach 1991. (DNMNH)

#### Hoplocoryphella grandis

Brancsik, 1895

D2591AAF-FD66-5AC0-A7B9-6A94BA22FEC9

##### Distribution

MDG, MAL, TZ, ZIM

##### Notes

ID: Dep. J.A.G. Rehn 1925 & A. Kaltenbach 1989. (DNMNH, IZIKO)

#### Hymenopoidea

Giglio-Tos, 1915

CBCD98D7-226D-5C4E-877B-E3BBF7B442B4

#### Empusidae

Burmeister, 1838

9383F994-8FD9-599B-A2BA-70BEAC26F72D

#### Empusinae

Burmeister, 1838

35DE2799-23EE-51D5-A5A4-174C8964D64E

#### Empusini

Ehrmann & Roy, 2002

8FD713F3-9F94-5D28-BD8D-E7B1EFF6944E

#### Empusina

Burmeister, 1838

B483AA33-E0CB-5F3A-8819-F72B8FF7653F

#### Empusa binotata

Audinet-Serville, 1839

3A65D15D-01E1-55BC-B22F-047048CB4851

##### Distribution

AL, AG, BF, CAM, ET, KN, LI, MDG, NAM, SM, TZ, CD, TU, IN

##### Notes

ID: Dep. R. Roy 1977, A. Kaltenbach 1998, M.B.D. Stiewe, R. Erhmann, C. Schwarz, A.J Hesse, H.D. Brown. (ARC, DNMNH, IZIKO, NMB, SMNK, PC_CS)

#### Idolomorphina

Ehrmann & Roy, 2002

43BC40F0-B8F3-521F-ABF0-B12EB547DAE0

#### Hemiempusa capensis

(Burmeister, 1838)

35F76711-3B34-5D2D-9A9A-2C29659E1BB2

##### Distribution

AG, GH, KN, DRC, RW, TZ, UG, ZIM

##### Notes

ID: Dep. J.A.G. Rehn 1916, R. Roy 1977, B.P. Uvarov & A.J. Hesse. (AMG, DNMNH, DNSM, IZIKO, NMSA, NRM)

#### Idolomorpha dentifrons

Zehntner & Saussure, 1895

0CFA80DF-0342-551E-A1BE-369A20CE2D4C

##### Distribution

ET, KN, MOZ, SM, SD, TZ, UG, ZB

##### Notes

ID: Dep. A.J. Hesse. (IZIKO)

#### Oxypilinae

Saussure, 1871

661DC8A8-433D-502B-BADB-B6D69BD08A33

#### Oxypilini

Saussure, 1871

3423C51E-254F-5B44-8540-1281C4990319

#### Junodia amoena

Schulthess-Rechberg, 1899

32A2EE28-F89A-51D7-8632-6E5EF0375921

##### Distribution

ET, KN, MOZ, TZ

##### Notes

ID: Undefined. (MNHN).

#### Junodia strigipennis

Westwood, 1889

9A2AD610-C982-5907-8B94-D6A5DA96C73E

##### Native status

Suspected to be endemic to southern Africa (Kaltenbach 1996)

##### Distribution

ET, MOZ, TZ, ZIM

##### Notes

ID: Dep. J.A.G. Rehn 1925 & R. Roy 1976. (DNMNH, IZIKO)

#### Junodia vansoni

Roy, 2009

4F8B3480-863A-5433-A3FB-C04A1E4FEB17

##### Distribution

TZ, MAL, ZAM

##### Notes

ID: Lit (Roy 2009)

#### Oxypilus (Anoxypilus) capensis

(Saussure, 1871)

1305ECF9-92B3-59EE-9498-960CE0D8F7DD

##### Distribution

AG, NAM, ZIM

##### Notes

ID: Dep. M. Beier 1952, R. Roy 1966; 1976, A. Kaltenbach 1984 & B.P. Uvarov. (ARC, DNMNH, IZIKO, NMSA)

#### Oxypilus (Anoxypilus) transvalensis

(Giglio-Tos, 1915)

32250FDC-DBAF-5F52-8272-7824B6A10C2E

##### Distribution

AG, BOT, ES, NAM, ZIM

##### Notes

ID: Dep. J.A.G. Rehn 1925, M. Beier 1952, R. Roy 1966 & A. Kaltenbach 1984. (DNMNH, IZIKO)

#### Hymenopodidae

Giglio-Tos, 1915

710CD776-9E7D-5F8D-9C73-E87B4FCE6689

#### Acromantinae

Brunner de Wattenwyl, 1893

1A936B59-2FC6-57A2-A371-09DD1747E97C

#### Otomantini

Giglio-Tos, 1915

6FE79EBB-48FF-589F-A536-49C5EB6AD687

#### Otomantis rendalli

Kirby, 1899

461134CB-EB3A-58C2-846D-A6A7E62C29A8

##### Native status

Suspected to be endemic to southern Africa (Kaltenbach 1996)

##### Distribution

ZAM

##### Notes

ID: Lit (Kaltenbach 1996, Ehrmann 2002

#### Otomantis scutigera

Bolivar, 1890

DEA8B047-5C6C-5547-BEE7-6926226E496F

##### Distribution

MAL, MOZ, TZ

##### Notes

ID: Dep. A.J. Hesse. (IZIKO)

#### Oxypiloidea (Oxypiloidea) tridens

Saussure, 1872

AC1F81DD-385D-523B-9B06-C946ED3E0FA3

##### Distribution

AG, CD, DRC, MOZ, NAM, TZ

##### Notes

ID: Dep. R.Roy 1988. (DNMNH, IZIKO)

#### Oxypiloidea (Oxypiloidea) namibiana

Roy, 2013

F86AF6A3-9041-58B4-B0C0-06BE8C7C9177

##### Distribution

BOT, ZIM, NAM

##### Notes

ID: Lit (Ehrmann 2002)

#### Hymenopodinae

Giglio-Tos, 1915

B4006B00-1255-540E-9B03-F0691253FD09

#### Hymenopodini

Giglio-Tos, 1915

397C9A9A-420A-52B6-BC5A-3721E3555928

#### Pseudocreobotrina

Brunner de Wattenwyl, 1893

25CA6A4E-41CD-58AA-9651-E16C8446A6C3

#### Pseudocreobotra wahlbergi

Stål, 1871

4775F71B-9718-5F8B-BA5E-1519F22E30B4

##### Distribution

AG, DRC, ET, KN, MAL, MOZ, TN, ZB, ZAM, ZIM

##### Notes

ID: Dep. J.A.G. Rehn 1925 & A. Kaltenbach 1998. (ARC, AMG, DNMNH, IZIKO, NRM)

#### Phyllocraniinae

Brunner de Wattenwyl, 1893

A678AD85-A010-5536-BA5A-E8B1780FAF58

#### Phyllocrania paradoxa

Burmeister, 1838

F8639AC1-E8C1-52B3-98C4-4460DE1C3DB5

##### Distribution

AG, CAM, DRC, ET, GH, GU, KN, MDG, MOZ, NAM, SM, SU, TZ, TG, UG, ZIM

##### Notes

ID: Dep. J.A.G. Rehn 1925, M. Beier 1952, R. Roy 1977, A. Kaltenbach 1985; 1988 & B.P. Uvarov. (AMG, ARC, DNMNH, IZIKO, NMSA, NRM)

#### Sibyllinae

Giglio-Tos, 1915

BB7C772C-D58A-56C3-8CEC-E30265EEE780

#### Sibylla pretiosa

Stål, 1856

316A7F7B-EC72-5B1E-A7F0-BB6DA57469C2

##### Distribution

DRC, ET, ES, KN, MAL, NAM, SM, TZ, UG, ZIM

##### Notes

ID: Dep. J.A.G. Rehn 1925, M. Beier 1952, R. Roy 1977, M.B.D. Stiewe & B.P. Uvarov. (ARC, DNMNH, NMSA, IZIKO, NMSA, NRM)

#### Mantoidea

Latreille, 1802

441D0EF6-41E1-5AD1-A0C8-82694E5C7282

#### Dactylopterygidae

Giglio-Tos, 1915

34C81073-D5C5-5AFD-9A7E-77711EBF17B6

#### Zouza radiosa

Giglio-Tos, 1907

BD2B1968-AA57-57C1-8245-C1D723AD3220

##### Distribution

BOT, NAM, ZAM, ZIM

##### Notes

ID: Dept. Kaltenbach 1988. (DNMNH)

#### Deroplatyidae

Latreille, 1802

B804B0AE-B9CB-5489-9971-C3E188A187B1

#### Popinae

Brunner de Wattenwyl, 1893

9486D421-2489-5AB8-9F0E-A64B533ABDD8

#### Popa spurca

Stål, 1856

8471ACC5-9B25-5F61-A409-DFC47E52F645

##### Distribution

AG, CAM, DRC, GH, GN, MOZ, NAM, TG, ZIM

##### Notes

ID: Dep. J.A.G. Rehn 1925, R. Roy 1977, B.P. Uvarov, & F. Werner. (AMG, DNMNH, IZIKO, EMÜ, NMSA, NMB, NRM)

#### Agrionopsis distanti

(Kirby, 1899)

89A59CB0-470D-5BF6-8A9F-3604E44C479F

##### Distribution

DRC, KN, UG, ZAM, ZIM

##### Notes

ID: Dep. J.A.G. Rehn 1923, H.D. Brown 1963, A. Kaltenbach 1991 & M. Beier. (ARC, DNMNH, IZIKO)

#### Danuria (Danuria) thunbergi

Stål, 1856

50DD8461-B792-5768-BCCF-4685617141D3

##### Distribution

DRC, ET, MOZ, NAM, TZ, ZIM

##### Notes

ID: Dep. Rehn 1926, A. Kaltenbach 1992, G.A.K. Marshall, A.J. Hesse, B.P. Uvarov, R. Erhmann & R. Roy. (DNMNH, IZIKO, NMSA, NRM, SMNK)

#### Mantidae

Latreille, 1802

87039709-1572-5D60-BA23-8954AA89B191

#### Mantinae

Latreille, 1802

E5C5301A-CAA0-524A-BA54-71182DE4AEB3

#### Mantis religiosa eichleri

Bazyluk, 1960

1875FA33-D6BF-5589-8E1B-C76BEBA2B29F

##### Distribution

Afro-Eurasia

##### Notes

ID: Dep. J.A.G. Rehn 1925, A. Kaltenbach 1985 & N. Moulin 2018. (ARC, BOLD, DNMNH, IZIKO, NMB, NHMUK)

#### Omomantinae

Giglio-Tos, 1916

FEA9D3C8-72A7-5E4F-997C-3F49B1871D26

#### Omomantis zebrata

Charpentier, 1843

B5B6B38D-F5D8-5485-8CE1-F284A207D80D

##### Distribution

KN, ZIM

##### Notes

ID: Dep. J.A.G. Rehn 1925, A. Kaltenbach 1985, M.B.D. Stiewe & B.P. Uvarov. (ARC, DNMNH, IZIKO, DNSM, NMSA, NRM)

#### Tenoderinae

Brunner de Wattenwyl, 1893

1851E75F-E555-59CE-B721-1743AAB34087

#### Paramantini

Roy, 1973

460FF68F-5954-52B5-938C-A47CCCB0D404

#### Paramantina

Roy, 1973

9CC07227-8771-5681-9FCC-8E8B38559B59

#### Paramantis natalensis

(Stål, 1856)

41D91293-ACE7-5A37-ABD8-1E24DAE5F2E0

##### Distribution

AG, CAM, DRC, ET, KN, TZ, ZIM

##### Notes

ID: Dep. R. Roy 1966 & A. Kaltenbach 1984. (DNMNH, NRM)

#### Paramantis sacra

Thunberg, 1815

A4E5F419-673F-561E-9EDD-7F090FC52AA0

##### Native status

Suspected to be endemic to southern Africa (Kaltenbach 1996)

##### Distribution

CAM, UG

##### Notes

ID: Dep. R.Roy & A. Kaltenbach 1991. (DNMNH, IZIKO, NRM)

#### Paramantis prasina

(Audinet-Serville, 1839)

BFAFC94C-CA05-541C-A4EA-674916D53951

##### Distribution

AG, GH, GN, KN, CAM, BG, TZ, TG

##### Notes

ID: Lit (Roy 1967, Ehrmann 2002)

#### Sphodromantis gastrica

Stål, 1858

CD74C3F4-9B49-5948-BF77-9CC375854B8C

##### Distribution

ET, MOZ, NAM, SM, TZ, UG, ZB, ZIM

##### Notes

ID: Dep. A. Kaltenbach 1984; 1988, N. Moulin 2018 & B.P. Uvarov. (ARC, AMG, DNMNH, NMSA, IZIKO, NMSA, NRM)

#### Sphodromantis gracilis

Lombardo, 1992

51E04EC7-7522-5821-8816-9D57C176A85E

##### Notes

ID: Lit (Ehrmann 2002)

#### Sphodromantis rudolfae

Rehn, 1901

1DA8FD46-7168-57FE-9FA2-538A3EFD478E

##### Distribution

ET, KN, SM, TZ, ZB

##### Notes

ID: Undefined. (NRM)

#### Tenoderini

Brunner de Wattenwyl, 1893

471ACB40-4834-5DFE-BCEF-B9D29332B59B

#### Polyspilotina

Giglio-Tos, 1917

FE10B0FA-0F21-57C6-8A3D-5786368F99FF

#### Polyspilota aeruginosa aeruginosa

(Goeze, 1778)

30723BF9-2C73-5066-BA34-5F024B0E59AD

##### Distribution

AG, CAM, CV, DRC, ET, GB, GN, KN, LB, MDG, NAM, SY, TZ, UG, ZB, ZIM

##### Notes

ID: J.A.G. Rehn 1925, M. Beier 1952, R. Roy 1977, Dep. A. Kaltenbach 1985; 1988, M.B.D. Stiewe & B.P. Uvarov. (ARC, BOLD, DNMNH, IZIKO, NMSA, NRM)

#### Polyspilota caffra

Westwood, 1889

65CC269F-8C4C-5037-9FFA-CF09245FFC4B

##### Native status

Suspected to be endemic to southern Africa (Kaltenbach 1996)

##### Distribution

ZIM

##### Notes

ID: Dep. J.A.G. Rehn 1925, R.Roy 1977 & C. Schwarz. (AMG, DNMNH, PC_CS)

#### Polyspilota magna

Giglio-Tos, 1911

9FCCF6E6-7AC3-5B32-AB87-C42E5FE61DBF

##### Native status

Suspected to be endemic to southern Africa (Kaltenbach 1996)

##### Notes

ID: Lit (Kaltenbach 1996, Ehrmann 2002)

#### Tenoderina

Brunner de Wattenwyl, 1893

F5508A23-60B7-5186-B122-75215DF13B5D

#### Epitenodera capitata

(Saussure, 1869)

78E0B9BA-2D1F-505F-9D22-5F8396767248

##### Distribution

AG, DRC, ET, KN, MAL, MOZ, UG, TZ, ZIM

##### Notes

ID: Dep. M. Beier 1952 & A. Kaltenbach 1992. (ARC, DNMNH, IZIKO)

#### Tenodera superstitiosa superstitiosa

(Fabricius, 1781)

F4511500-8E9C-5F8C-A294-1C7B986A4E39

##### Distribution

AG, AZ, CAM, DRC, ET, GB, GH, GN, IN, JV, KN, LB, MAL, MOZ, NAM, NG, SG, SM, SD, TZ, TG, UG, ZAM, ZB

##### Notes

ID: Dep. M. Beier 1952 & A. Kaltenbach 1985. (DNMNH, IZIKO)

#### Miomantoidae

Westwood, 1889

C303DDDB-91FC-56F6-9005-07653607F777

#### Miomantidae

Westwood, 1889

9B535FCB-D8C0-5A26-A4CF-B4C05E8CF5D3

#### Miomantinae

Westwood, 1889

8CC7CC5E-62BF-5B70-ADEA-A54A7789385F

#### Cilnia chopardi

Werner, 1927

734768DC-E7EA-59B3-8761-045558E2D5FA

##### Distribution

BOT, MOZ, NAM

##### Notes

ID: Dep. M. Beier 1952. (DNMNH)

#### Cilnia humeralis

(Saussure, 1871)

4A5A4DBB-9B8A-50B1-87DA-0CA658207228

##### Distribution

AG, DRC, MAL, MOZ, TZ, ZAM, ZIM

##### Notes

ID: Dep. Rehn 1925, A. Kaltenbach 1985, A.J. Hesse, J.A.G. J.A.G. Gain, R. Ehrmann, B.P. Uvarov & R. Roy. (DNMNH, IZIKO, NMSA, NMB, SMNK, PC_CS)

#### Miomantis aequalis

Rehn, 1904

A7AD0C8B-83BC-59A3-8969-CD3EBCFADB90

##### Native status

Suspected to be endemic to southern Africa (Kaltenbach 1996)

##### Notes

ID: Dep. J.A.G Rehn 1925. (DNMNH)

#### Miomantis binotata

Giglio-Tos, 1911

5050E2D2-B5BD-5C7F-A646-F249F2EE5F26

##### Distribution

KN, MAL, RW, TZ, TG

##### Notes

ID: Lit (Ehrmann 2002)

#### Miomantis brachyptera

Saussure, 1899

551B020A-4ED8-513A-9399-AE5405AF2D3D

##### Notes

ID: Lit (Ehrmann 2002)

#### Miomantis brevipennis

Saussure, 1872

F85C2898-22BA-54D8-A047-5F72CCFBBD63

##### Distribution

CAR, ET, KN, DRC, TZ, ZB

##### Notes

ID: Lit (Ehrmann 2002)

#### Miomantis caffra

Saussure, 1871

AD138BED-FEB2-57D8-A697-D4BFBB008E3B

##### Native status

Suspected to be endemic to southern Africa (Kaltenbach 1996)

##### Distribution

EW, NZ

##### Notes

ID: Dep. J.A.G. Rehn 1925, A. Kaltenbach 1992, R. Roy & C. Schwarz. (DNMNH, PC_CS)**

#### Miomantis coxalis

Saussure, 1898

16DF31FE-766D-5B0A-8CD6-2C8122B26C8C

##### Distribution

AG, LS, MOZ, NAM

##### Notes

ID: Dep. J.A.G. Rehn 1925, M. Beier 1952 & A. Kaltenbach 1988. (DNMNH, IZIKO)

#### Miomantis exilis

Giglio-Tos, 1911

ED7174B9-B707-53BA-9843-9D00612004E9

##### Distribution

AG, BOT, ET, SM, NAM

##### Notes

ID: Dep. J.A.G. Rehn 1925, M. Beier 1952 & A. Kaltenbach 1988. (DNMNH, IZIKO)

#### Miomantis fenestrata

Fabricius, 1781

ECB7DBBD-2503-52D0-84BB-34A3494CD526

##### Native status

Suspected to be endemic to southern Africa (Kaltenbach 1996)

##### Distribution

DRC, LS, MOZ, NAM, SM, UG

##### Notes

ID: Dep. J.A.G. Rehn 1925, A. Kaltenbach 1988 & B.P. Uvarov. (ARC, DNMNH, IZIKO, NMSA, NMB, NRM)

#### Miomantis helenae

Giglio-Tos, 1914

1107CCE0-1EF0-5CF0-A9B3-627A0A2A196F

##### Distribution

MAL, ZIM

##### Notes

ID: Dep. J.A.G. Rehn 1925 & A. Kaltenbach 1988. (DNMNH)

#### Miomantis kibweziana

Giglio-Tos, 1911

E3BFBCA9-4DAF-5B33-A3EF-BA23AB595F8C

##### Distribution

KN, TZ, UG

##### Notes

ID: Dep. R. Roy. (NRM)

#### Miomantis lacualis

Giglio-Tos, 1911

4524C084-9F3B-5A44-8E33-9AD48CA20F70

##### Distribution

ET, KN, MAL,MOZ,SM,TZ,UG

##### Notes

ID: Lit (Kaltenbach 1996, Ehrmann 2002)

#### Miomantis minuta

Giglio-Tos, 1911

9E5BAF98-8083-5300-B338-4A617413B279

##### Native status

Suspected to be endemic to southern Africa (Kaltenbach 1996)

##### Notes

ID: Unspecified. (NRM)

#### Miomantis misana

(Giglio-Tos, 1911)

6080F0FB-A692-5C04-8543-733BDDCF3203

##### Distribution

AG, CAM, DRC, GN, TG

##### Notes

ID: Undefined. (NRM)

#### Miomantis monacha

Fabricius, 1787

7DA23746-7C8A-5360-87A3-C3B2F1210E43

##### Distribution

MOZ

##### Notes

ID: Lit (Ehrmann 2002)

#### Miomantis natalica

Beier, 1930

2A385CA2-3ED5-5B8A-B951-D3843F45F43E

##### Native status

Suspected to be endemic to southern Africa (Kaltenbach 1996)

##### Distribution

NAM, ZIM

##### Notes

ID: Dep. A. Kaltenbach 1988; 1992. (AMG, DNMNH)

#### Miomantis paykullii

Stål, 1871

97051BD9-96DC-5AB3-A086-6D1354E4C9B6

##### Distribution

EG, BF, GH, CAM, KN, MOZ, NG, SG, TG, TC, UG, ZIM

##### Notes

ID: Undefined. (NRM)

#### Miomantis prasina

Burmeister, 1838

B3789B96-6080-5A1A-8787-03D97487C4A0

##### Distribution

AG, DRC, MOZ

##### Notes

ID: Dep. A. Kaltenbach 1981. (DNMNH)

#### Miomantis preussi

Karsch, 1892

BA6AAD69-EC60-5F8E-8422-5545E212FC0D

##### Distribution

CAM

##### Notes

ID: F. Werner. (NRM)

#### Miomantis quadripunctata

Saussure, 1898

E5F826BD-6C06-52B7-BF48-746BA87CFE07

##### Distribution

DRC, MOZ, NAM, TZ, UG, ZIM

##### Notes

ID: Dep. A. Kaltenbach 1988; 1992, R. Ehrmann 1991, B.P. Uvarov & F. Werner. (ARC, DNMNH, IZIKO, NMSA, NRM)

#### Miomantis saussurei

Schulthess-Rechberg, 1899

064A9A4E-B388-5BF4-AE62-45ABE07B45E7

##### Distribution

MOZ, SM

##### Notes

ID: Dep. J.A.G. Rehn 1925. (DNMNH, IZIKO, NRM)

#### Miomantis semialata

Saussure, 1872

7912D2B7-8FE4-5D13-B10F-C7D07D0936CB

##### Native status

Suspected to be endemic to southern Africa (Kaltenbach 1996)

##### Distribution

EW, NAM, TZ, ZIM

##### Notes

ID: Dep. M. Beier 1952 & A. Kaltenbach 1988; 1992. (DNMNH, IZIKO, NRM)

#### Neocilnia gracilis

Beier, 1930

26CD8808-25EB-5309-B4E7-BFF4A74BD1D4

##### Native status

Suspected to be endemic to southern Africa (Kaltenbach 1996)

##### Notes

ID: N. Moulin 2018. (DNMNH)

#### Parasphendale costalis

Kirby, 1904

D1C7D487-A7E9-5782-B9CB-4D9116718563

##### Distribution

ET, KN, SM, TZ, UG, ZIM

##### Notes

ID: Lit (Ehrmann 2002)

#### Taumantis globiceps

Beier 1969

CA1115F4-1460-56D0-A0AB-79AA1C8338C5

##### Distribution

MAL, ZAM, ZIM

##### Notes

ID: Dep. R. Ehrmann 1991. (ARC)

#### Solygiinae

Giglio-Tos, 1919

AFB8A5C1-725A-5FF6-9B05-C18D14E71F15

#### Solygia sulcatifrons

Audinet-Serville, 1839

5E140BA5-DDE4-57B0-98EC-611DCC840E56

##### Distribution

BF, CAM, GN, SN, TG

##### Notes

ID: Dep. B.P. Uvarov. (NMSA, ARC)

#### Nanomantoidea

Brunner de Wattenwyl, 1893

B5BE9AEF-81FD-54E5-8007-5E5E893E4999

#### Amorphoscelidae

Stål, 1877

092AB799-71CD-5682-B624-F10BF3DAAD00

#### Amorphoscelinae

Stål, 1877

34485786-9C4C-555E-913E-F2361771B4A6

#### Amorphoscelis austrogermanica

Roy, 1963

A585B89C-F895-5A8E-9389-E10D018976F6

##### Distribution

East Africa, NAM

##### Notes

ID: Dep. R. Roy 1967 & & R. Erhmann. (DNMNH, SMNK)

#### Amorphoscelis tuberculata

Werner, 1923

4AB49BF7-022E-57A6-83DE-219FBE542FD2

##### Distribution

MOZ, NAM, ZIM

##### Notes

ID: Dep. R. Roy 1962 & R. Erhmann. (DNMNH, SMNK)

#### Hapalomantinae

Beier, 1962

7EC2C2DD-DC40-527A-A1E0-22A7DCF93EAE

#### Hapalomantini

Beier, 1962

7A790F10-3C90-50FC-A67D-B3924DA072C6

#### Hapalogymnes gymnes

Rehn, 1927

0EDAFC9A-BE86-58AF-B368-B6339A329CEB

##### Native status

Suspected to be endemic to southern Africa (Kaltenbach 1996)

##### Distribution

ZIM

##### Notes

ID: Dep. J.A.G. Rehn 1925. (DNMNH)

#### Bolbena (Bolboda) minutissima

(Karny, 1908)

8B725E41-8E5D-52BB-9327-0E754F6DB1C6

##### Native status

Suspected to be endemic to southern Africa (Kaltenbach 1996)

##### Distribution

ZIM

##### Notes

ID: Dep. M. Beier 1952. (DNMNH, IZIKO)

#### Hapalomantis (Bolbira) minima

(Werner, 1906)

6818778F-137A-5D8B-AA7A-2224D8B1814C

##### Distribution

AG, ZIM

##### Notes

ID: Lit (Kaltenbach 1996, Ehrmann 2002)

#### Hapalomantis (Hapalomantis) orba

(Stål, 1856)

C0999094-B237-5088-92DA-E8A068987FF1

##### Distribution

KN, MOZ, TZ

##### Notes

ID: Dep. M. Beier 1953 & A.J. Hesse. (BOLD, DNMNH, IZIKO, NRM)

#### Nilomantini

Ehrmann & Roy, 2002

EBAFA9BD-A226-5A44-A152-66AE88E84E66

#### Chloromantis rhombica

Giglio-Tos, 1915

C1958930-DCC6-5A13-8DD5-474895555998

##### Distribution

ZIM

##### Notes

ID: Lit (Kaltenbach 1996, Ehrmann 2002)

#### Negromantis gracillima

Kaltenbach, 1996

8387DB04-A76F-5DFD-9E79-EE8B632910B0

##### Distribution

ZAM, ZIM

##### Notes

ID: Dep. A. Kaltenbach. (IZIKO)**

## Analysis

**Figure 3.**
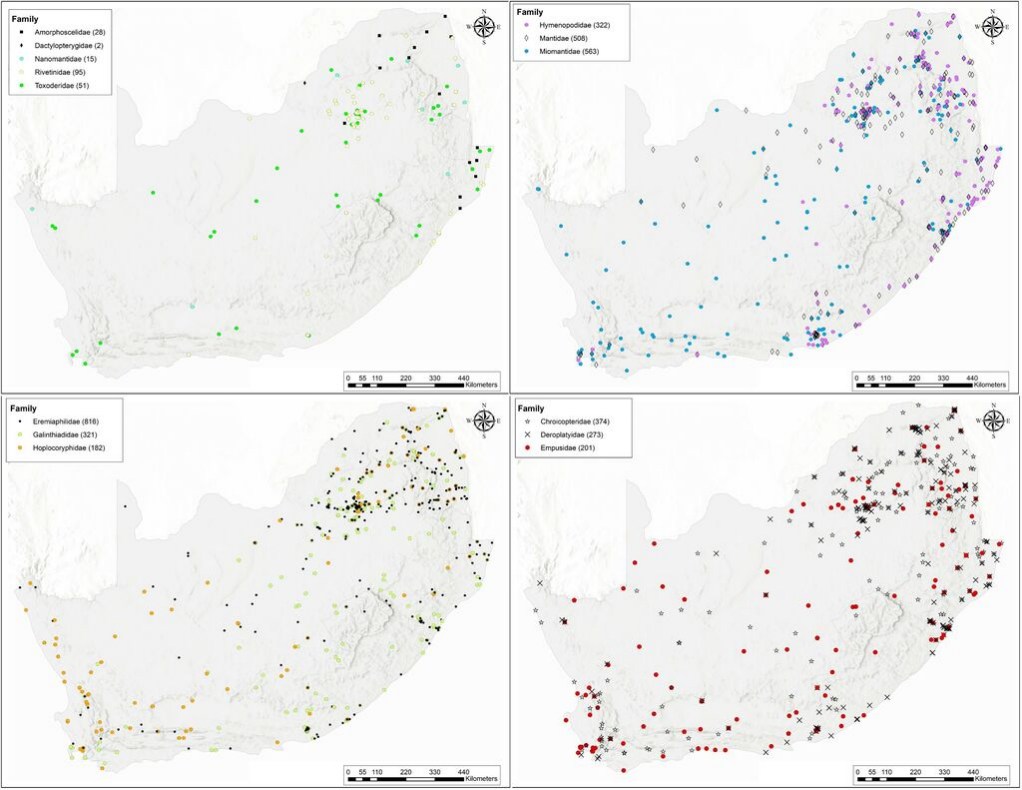
Distribution maps of the Mantodea families that occur within South Africa. The number of specimen records per family is provided in brackets.

This checklist was compiled from a database that has been generated after recording all the available details of specimens (approximately 4000 records over 170 years) in eight South African museum collections. An additional 1945 Mantodea records from private collections, several museums outside of South Africa, and two citizen-science platforms were included. Although all specimens were identified to family level, a large number (1600) were only identified to genus level. All records within the database could, therefore, be used to generate distribution maps for the 14 Mantodea families (Fig. 3). Despite only a few distribution records being available for specimens of some of the families, distribution patterns indicate that all families occur in the hotspots indicated in this study. These hotspots are the north-eastern parts of the Savannah biome (towards the Kruger National Park), along the eastern coast in the Indian Ocean coastal belt (KwaZulu-Natal province), southern coastal region in the thicket biome (Eastern Cape – Gqeberha), and fynbos biome in the south-western Cape region. The families with the lowest species richness and the lowest number of records were Amorphoscelidae, Dactylopterygidae, Nanomantidae, Rivetinidae and Toxoderidae (Fig. 3). The three most species-rich families were Mantidae, Miomantidae and Eremiaphilidae (Fig. 3), which were also the families with the highest numbers of specimen records. The latter three families made up 50.2% of the total number of specimens in the surveyed collections, with those in the Eremiaphilidae having the highest representation (853 specimens). The distribution maps do, however, elude that some areas of the country have either been under-represented or the abundance of mantids in these regions, for example the Northern Cape Region, is very low. Interestingly, the distribution of Empusidae, which is only represented by seven species, indicates a region-wide distribution and more than 200 records of this family were recorded during this study.

## Discussion

This paper illustrates the value of museum data, although it was only after the documentation thereof, that these data allowed us to update the Mantodea species list of South Africa. Historic data encompasses many years of collected specimens and, as suggested by Hill et al. (2012), it remains as data that are invaluable and irreplaceable. Although the more modern methods of observational data collection through citizen-science platforms are easily accessible (Moulin 2020), it remains problematic as the identification of these specimens are difficult, especially for groups such as arthropods. This is due to the vast numbers of insect species and because, in many cases, identification requires microscopic investigation of the genitalia and wing venation. Due to these difficulties, as well as data only at genus level, records sourced from the citizen-science platforms were only used to compile the distribution maps and they were not included in the supplementary information. Although citizen-science platforms may provide valuable information (Moulin 2020), care should be taken when data are used. For example, 11 *Miomantis* species were recorded from the museum collections during this study, while nine other species are also suggested to occur in South Africa (Kaltenbach 1996, Kaltenbach 1998). However, all 467 *Miomantis* observations from iNaturalist and GBIF.org were listed as *Miomantiscaffra* Saussure, 1871. Since no other *Miomantis* species has been recorded on the latter platforms, it is highly likely that some of the *Miomantis* species level identifications were incorrect. Despite this type of error, the data recorded on citizen-science platforms can be helpful to determine distribution patterns. These platforms are becoming increasingly important, but there is no substitution for taxonomic expertise and investigation of specimens, especially for the many species of Mantodea that have not yet been added to publicly accessible DNA databases. For example, of the 157 species listed in this checklist, only 50 are represented on DNA databases such as GenBank, NCBI and BOLD.

Another example which illustrates the value of data from citizen-science platforms (Gbif.org in this case), as well as the caution needed in interpreting such data, is that of *Pseudocreobotraocellata*. This species, according to literature, is native to North Africa and does not occur in South Africa (Ehrmann 2002). However, a DNA barcode of the latter species was found on the BOLD database (Ratnasingham and Hebert 2007) during this study which, according to the locality of the specimen used for the DNA analysis, it was collected in KwaZulu-Natal in South Africa (Suppl. material 1). The sequence provided on BOLD is 99.23% similar to that of an unpublished DNA sequence of *Pseudocreobotrawahlbergi* that is available on GenBank. The latter sequence has never been published and should, thus, be treated with caution. The specimens identified as *P.occellata* on the BOLD system could either be new distribution records or be due to a lack of reliable *P.wahlbergi* sequences.

One of the species, *Galepsuscentralis*, which has not previously been reported from South Africa, was collected in the Grassland biome in the north-western region of South Africa and identified by a mantid taxonomist (Nicolas Moulin) during this study. This species was previously reported to occur only in Tanzania and the Democratic Republic of the Congo. It could also be possible that this species has always occurred in southern Africa, but was not detected or it has expanded its range to southern Africa, similar to what has been reported for other mantid species in recent years (Schwarz and Ehrmann 2018). Interestingly, Kaltenbach (1996) listed some species that may be endemic to southern Africa, including *Miomantiscaffra*. The latter species has expanded its range to New Zealand and Australia (Ramsay 1984, Connors et al. 2022).

This species list indicates that the diversity of Mantodea in South Africa is high and that approximately 6% of the known Mantodea species worldwide occurs in this region. A few areas within South Africa seem to be “hotspots” or regions with high diversity and should be investigated further. These areas may be related to the biomes within the country since insect communities tend to be closely correlated to plant communities (Schaffers et al. 2008). It is, therefore, suggested that the “hotspot” areas identified in this study be priority areas for future research. The conglomeration of distribution records on the maps in areas such as Pretoria may be due to the ease of access (Grytnes and Romdal 2008) of this museum as it resides within a large city with a large surrounding human population.

The current state of knowledge suggests that South Africa could have a high level of Mantodea endemicity, i.e. 38% of the species from this study are suggested by Kaltenbach (1996) to be endemic to southern Africa. Furthermore, "priority species" (for example, the 24 species of which only one record exists) were identified and should be investigated first to address the severe lack of knowledge regarding these species. For example, *Calamothespisoxyops* has a single distribution record (Baberton 1910), i.e. the Holotype specimen in the Ditsong National Museum of Natural History. One other record of this species was recorded on iNaturalist (which could indicate that it only occurs in South Africa). This citizen-science record has, however, not been verified (not Research Grade) and was, thus, not included in the Suppl. material 1. Lastly, the aim of this paper was not only to update the Mantodea checklist of South Africa, but also to develop a dataset that can guide future research on Mantodea diversity within the region. The absence of taxonomic expertise to identify Mantodea in South Africa provided a challenge during the compilation of this checklist. This was addressed through collaboration with the international Mantodea scientist community who assisted with identifications, provided taxonomic keys and shared old literature regarding the Mantodea of the region.

## Supplementary Material

2E983403-0412-5918-9B19-5F46CFC604EB 10.3897/BDJ.11.e102637.suppl1 Supplementary material 1 Database of all Mantodea records within South Africa Data type occurrences, taxonomic Brief description This file contains the 5945 records of Mantodea that have been recorded to occur in South Africa – Each record has locality, taxonomic, collectors etc. data. This database is a compilation of records collected during visits to museums, as well as online records such as Gbif.org and iNaturalist. Prominent European and American museums were also contacted to provide information on the South African Mantodea fauna in their collections. File: oo_927343.xlsx https://binary.pensoft.net/file/927343 B Greyvenstein, H Du Plessis, J van den Berg

F5F8E083-C284-53AA-A259-B0E40FF6BE22 10.3897/BDJ.11.e102637.suppl2 Supplementary material 2 Number of species and records per taxonomical hierarchy of Mantodea in South Africa Data type Species data Brief description This spreadsheet contains a summation of the number of records per superfamily, family, subfamily etc. of Mantodea that were recorded during this study from various institutions and collections. It also includes the number of species per taxonomic level and the numbers of different specimen types, for example Holotype or Paratype, that were recorded during this study. File: oo_927344.xlsx https://binary.pensoft.net/file/927344 B Greyvenstein, H Du Plessis, J van den Berg

## Figures and Tables

**Table 1.** List of abbreviations for the museums and collections in which the Mantodea specimen are hosted.

Institution/ Museum collection	Abbreviation
Agricultural Research Council Roodeplaat, Pretoria, SA	ARC
Albany Museum, Makhanda (Grahamstown), SA	AMG
Barcode of life data system, availible online	BOLD
Cleveland Museum of Natural History, Ohio, USA	CMNH
Ditsong National Museum of Natural History, Pretoria, SA	DNMNH
Durban Natural Science Museum, Durban, SA	DNSM
Estonian University of Life Sciences, Institute of Agricultural and Environmental Sciences, Tartu, Estonia	EMÜ
Iziko South African Museum, Cape Town, SA	IZIKO
KwaZulu-Natal Museum, Pietermaritzburg, SA	NMSA
Lund University Biological Museum, Lund, Sweden	MZLU
Muséum national d'Histoire naturelle, Paris, France	MNHN
National Museum, Bloemfontein, SA	NMB
Natural History Museum, London, UK	NHMUK
Personal collection of Christian Schwarz, Germany	PC_CS
Personal collection of Johnnie van den Berg, SA	PC_JB
Staatliches Museum für Naturkunde, Karlsruhe, Germany.	SMNK
Student Collection, Rhodes University, Makhanda (Grahamstown), SA	RU
Swedish Museum of Natural History, Stockholm, Sweden	NRM

**Table 2.** Abbreviations of country names listed in the section describing the distribution of different Mantodea species recorded in South Africa.

Abbreviation	Country	Abbreviation	Country	Abbreviation	Country
[AL]	Algeria	[GN]	Guinea	[NG]	Nigeria
[AG]	Angola	[IN]	India	[RNI]	Rennell Island
[AZ]	Australia	[IS]	Israel	[RI]	Reunion Island
[BA]	Bismarck Archipelago	[IC]	Ivory Coast	[RW]	Rwanda
[BOT]	Botswana	[JP]	Japan	[SG]	Senegal
[BF]	Burkina Faso	[JV]	Java	[SY]	Seychelles
[BR]	Burundi	[KN]	Kenya	[SL]	Sierra Leone
[CAM]	Cameroon	[LS]	Lesotho	[SM]	Somalia
[CA]	Canada	[LB]	Liberia	[SD]	Sudan
[CV]	Cape Verde	[LI]	Libya	[TZ]	Tanzania
[CAR]	Central African Republic	[MDG]	Madagascar	[TH]	Thailand
[CD]	Chad	[MAL]	Malawi	[TG]	Togo
[CH]	China	[MP]	Malayan Peninsula	[TC]	Tschad
[DRC]	Dem. Rep. Congo	[MA]	Mali	[TU]	Tunisia
[EG]	Egypt	[MN]	Mauritania	[UG]	Uganda
[EW]	Eswatini	[MT]	Mauritius	[UK]	United Kingdom
[ET]	Ethiopia	[MOZ]	Mozambique	[ZAM]	Zambia
[GB]	Gabon	[NAM]	Namibia	[ZB]	Zanzibar
[GH]	Ghana	[NU]	New Guinea	[ZIM]	Zimbabwe
[GU]	Guam	[NZ]	New Zealand		

## References

[B10536180] Connors Matthew G., Chen Honglei, Li Haokun, Edmonds Adam, Smith Kimberley A., Gell Colin, Clitheroe Kelly, Miller Ishbel Morag, Walker Kenneth L., Nunn Jack S., Nguyen Linh, Quinane Luke N., Andreoli Chiara M., Galea Jason A., Quan Brendon, Sandiford Katrina, Wallis Brendan, Anderson Matthew L., Canziani Elizabeth Valeria, Craven Jade, Hakim Roi R. C., Lowther Rod, Maneylaws Cindy, Menz Bastian A., Newman John, Perkins Harvey D., Smith Alistair R., Webber Vanessa H., Wishart Dylan (2022). Citizen scientists track a charismatic carnivore: Mapping the spread and impact of the South African Mantis (Miomantidae, *Miomantiscaffra*) in Australia. Journal of Orthoptera Research.

[B8809643] Ehrmann R. (2002). Mantodea: Gottesanbeterinnen der Welt.

[B8809651] Green T. (2014). Praying mantis: Ultimate care guide.

[B10530829] Grytnes J., Romdal T. S. (2008). Using museum collections to estimate diversity patterns along geographical gradients.. Folia Geobotanica.

[B8809659] Hill Andrew, Guralnick Robert, Smith Arfon, Sallans Andrew, Gillespie Rosemary, Denslow Michael, Gross Joyce, Murrell Zack, Conyers Tim, Oboyski Peter, Ball Joan, Thomer Andrea, Prys-Jones Robert, de la Torre Javier, Kociolek Patrick, Fortson Lucy (2012). The notes from nature tool for unlocking biodiversity records from museum records through citizen science. ZooKeys.

[B8809680] Kaltenbach A. P. (1996). Unterlagen für eine Monographie der Mantodea des südlichen Afrika: 1. Artenbestand, geographische Verbreitung und Ausbreitungsgrenzen (Insecta: Mantodea). Annalen des Naturhistorischen Museums in Wien.

[B8809689] Kaltenbach A. P. (1998). Unterlagen für eine Monographie der Mantodea (Insecta) des südlichen Afrika: 2. Bestimmungstabellen für die höheren Taxa, Nachträge zum Artenbestand. Annalen des Naturhistorischen Museums in Wien.

[B8809698] McMonigle O. (2013). Keeping the praying mantis.

[B10619513] Moulin Nicolas (2020). When Citizen Science highlights alien invasive species in France: the case of Indochina mantis, *Hierodulapatellifera* (Insecta, Mantodea, Mantidae). Biodiversity Data Journal.

[B8809715] Otte D, Spearman L, Stiewe MBD Mantodea Species file online. Version 5.0/5.0.. http://Mantodea.SpeciesFile.org..

[B10536171] Ramsay G. W. (1984). *Miomantiscaffra*, a new mantid record (Mantodea: Mantidae) for New Zealand. New Zealand Entomologist.

[B10536132] Ratnasingham Sujeevan, Hebert PAUL D. N. (2007). BARCODING: bold: The Barcode of Life Data System (http://www.barcodinglife.org). Molecular Ecology Notes.

[B10869104] Roy R. (1967). Contribution à la connaissance des genre Mantis Linne et Paramantis, nov. [Mantidae]. Mantidae). Bulletin de l'Institut Fondamental d'Afrique Noire (IFAN), Série A.

[B10536123] Roy R (2004). Rearrangements critiques dans la famille des Empusidae et relations phylogenetique [Dictyopera, Mantodea]. Association des Amis du Laboratoire d'Entomologie du Muséum, Paris.

[B8809723] Roy Roger (2006). Deux nouvelles synonymies au niveau genre (Dictyoptera, Mantodea). Bulletin de la Société Entomologique de France.

[B10536105] Roy Roger (2009). Nouvelles données sur le genre *Junodia* Schultess, 1899 (Mantodea, Hymenopodidae). Bulletin de la Société Entomologique de France.

[B8809732] Roy Roger (2010). Mises au point sur le genre *Sphodromantis* Stål, 1871 (Mantodea, Mantidae). Bulletin de la Société Entomologique de France.

[B10536114] Roy Roger (2013). Révision du genre africain Oxypiloidea Schulthess, 1898 (Dictyoptera, Mantodea, Hymenopodidae). Zoosystema.

[B8809741] Roy R. (2018). Le genre *Tenodera* Burmeister, 1838, généralités et présence en Afrique (Mantodea, Mantidae). Bulletin de La Société Entomologique de France.

[B8809750] Roy Roger (2022). Révision du genre afrotropical *Epitenodera* Giglio-Tos, 1912 (Mantodea, Mantidae). Bulletin de la Société Entomologique de France.

[B10530882] Schaffers André P., Raemakers Ivo P., Sýkora Karlè V., ter Braak Cajo J. F. (2008). Arthropod assemblages are best predicted by plant species composition. Ecology.

[B8809759] Schoeman AS (1985). Hottentotsgotte en Stokinsekte.

[B8809767] Schoeman AS, Scholtz HC, Holm E (1985). Insects of Southern Africa.

[B10536141] Schwarz C., Ehrmann R. (2018). Invasive Mantodea species in Europe. Articulata.

[B8809780] Schwarz Christian J., Roy Roger (2019). The systematics of Mantodea revisited: an updated classification incorporating multiple data sources (Insecta: Dictyoptera). Annales de la Société entomologique de France (N.S.).

[B8809789] Svenson GJ, Wieland F, Rivera J, Tedrow R, Brannoch SK, Rodriques HM, MIller K, Grimaldi D Project Mantodea: Systematics and evolution. https://mantodearesearch.com/.

[B8809801] Wieland F., Schutte K. (2012). McGraw-Hill Encyclopedia of Science & Technology: Mantodea.

[B8809809] Wieland Frank (2013). The phylogenetic system of Mantodea (Insecta: Dictyoptera). Georg-August-University Göttingen.

